# Is higher resilience predictive of lower stress and better mental health among corporate executives?

**DOI:** 10.1371/journal.pone.0218092

**Published:** 2019-06-11

**Authors:** Cindy A. Kermott, Ruth E. Johnson, Richa Sood, Sarah M. Jenkins, Amit Sood

**Affiliations:** 1 Division of General Internal Medicine, Mayo Clinic, Rochester, Minnesota, United States of America; 2 Division of Preventive Medicine, Mayo Clinic, Rochester, Minnesota, United States of America; 3 Division of Biostatistics and Informatics, Mayo Clinic, Rochester, Minnesota, United States of America; Chiba Daigaku, JAPAN

## Abstract

**Objective:**

To assess the impact of resilience, the ability to withstand and bounce back from adversity, on measures of well-being, self-reported stress, and mental health diagnoses.

**Methods:**

This study was a cross-sectional survey of participants seen at an executive health practice at Mayo Clinic, Rochester, Minnesota, from January 2012 through September 2016. Participants completed an anonymous survey that included demographic information and 3 validated survey instruments—the 10-item Connor-Davidson Resilience Scale (CD-RISC), the 12-item Linear Analogue Self-Assessment Scale (LASA), and the 14-item Perceived Stress Scale (PSS). Self-reported history of mental health diagnoses was also collected. CD-RISC scores were used to stratify participants into lower (<30), medium (30–34), or higher (≥35) resilience categories. Participants’ LASA scores, PSS scores, and self-reported mental health diagnoses were compared among resilience categories.

**Results:**

Of the 2,027 eligible participants, 1,954 met the study inclusion criteria as currently employed corporate-sponsored executive or business professionals (self-designated) who completed the CD-RISC survey. Most participants (62.5%) were aged 40 to 59 years. The majority were male (78.3%), white (95.3%), educated (86.2%), and in a committed relationship (89.7%). Among participants, 41.7% reported higher resilience, 34.3% had medium resilience, and 24.0% had lower resilience. The quality of life and overall LASA scores were positively associated with higher resilience (*P* < .001). PSS scores and self-reported mental health diagnoses were negatively associated with higher resilience (*P* < .001). These associations remained significant after adjusting for patient characteristics.

**Conclusions:**

In this cross-sectional survey of a large cohort of corporative executives, the lower-resilience cohort had a 4-fold higher prevalence of depression and an almost 3-fold higher prevalence of anxiety compared with the higher-resilience cohort. High resilience was positively associated with well-being and negatively associated with perceived stress. Our findings suggest that higher resilience in the executive workplace environment is associated with better mental health, reduced stress, and greater well-being.

## Introduction

The World Health Organization has declared stress as the global epidemic of the 21st century. Approximately 80% of US workers now report feeling stressed at the workplace [1]. Commonly cited reasons for greater stress are increasing workload, interpersonal issues, imbalance between personal and work lives, adverse working conditions, and lack of job security [1–4]. The corporate world is subject to stress from economic pressures, competition, long working hours, downsizing, tight budgets, overall uncertainty, lack of support, unfair treatment, low decision latitude, conflicting roles, poor communication, a low sense of contribution to the society, gender inequality, and workplace bullying [3, 5–7].

Work stress is also a known risk factor for occupational burnout, depression, anxiety, and suicide [8–11]. Occupational stress affects musculoskeletal health (eg, back pain, neck pain, fatigue), increases risk of cardiovascular disease, is a risk factor for diabetes mellitus, stroke, and dementia, contributes to accidents, absenteeism, turnover, and lower productivity, and increases medical, legal, and insurance costs [1, 12–21]. The estimated cost of stress to US businesses is $300 billion annually [1].

An increasingly recognized protective factor against stress is resilience. Resilience is defined as one’s ability to bounce back from adversity and view adversity as an opportunity for growth [22]. Although a few previous studies have evaluated the association of resilience with lower stress and better mental health [23], the effect of resilience in reducing workplace stress and mental health in the corporate setting has not been well studied. A few studies, mostly of nurses working in health care settings, showed a positive correlation between resilience and the ability to bounce back after a workplace conflict [24], a negative correlation between resilience and burnout [25], and higher job satisfaction with high self-reported resilience [26]. Resilience is also correlated with buffering of workplace stress and adverse mental health outcomes among critical care professionals [27] and with better work satisfaction among physicians [28].

Workers, particularly corporate executives, are an understudied group in terms of the effects of resilience. An Australian study showed that positive mental health mitigated the effect of workplace stress on personal feelings of distress [29]. Another study used an online survey−based proprietary tool to assess workers and showed that resilience had a protective effect on stress, burnout, job satisfaction, intention to quit, likelihood of absence, productivity loss, sleep problems, and likelihood of depression [30]. Nevertheless, data about the impact of resilience on mental health, stress, and well-being measures among corporate executives is currently sparse. The present study was designed to assess the association of resilience with self-reported measures of stress and well-being and self-reported mental health diagnoses by surveying a large number of corporate executives participating in an executive health practice.

## Methods

### Ethical considerations

The study was approved by the Mayo Clinic Institutional Review Board (protocol 11–000527) and adhered to the principles described in the Declaration of Helsinki. Informed verbal consent was obtained from study participants. Participants were notified that their participation was voluntary and had no impact on their clinical care. No payment or remuneration was offered as a result of participation. The study excluded minors and respondents who were not business executives or other professionals. The reporting of this study is in compliance with the STROBE (Strengthening the Reporting of Observational Studies in Epidemiology) statement [31].

### Study design

The study was designed as a cross-sectional survey of participants of the Executive Health Program at Mayo Clinic (Rochester, Minnesota) from January 1, 2012, through September 30, 2016. The Executive Health Program at Mayo offers a comprehensive, preventive medical evaluation and serves to provide focused access to health care. This program primarily serves busy executives, business and other professionals, their spouses, and others who choose (self-select) to have this level of service.

### Survey administration

Potential participants received an introductory letter that detailed the study aims, provided information about the study risks and benefits, and indicated the time needed to complete the survey (approximately 30 minutes). Surveys were distributed by clinical assistants at the first contact with participants during the check-in process. Participants were asked if they had previously completed the survey, and they were requested to decline participation if they had taken the survey earlier. Participants completed the survey while waiting for their clinical appointments. Completed surveys were deposited into a locked collecting receptacle in the waiting lounge; surveys were collected on a weekly basis. Survey data were entered into an electronic database using the (Research Electronic Data Capture (REDCap) data entry system [32].

### Survey instrument

The survey instrument consisted of greater than 200 items developed in collaboration with Mayo’s Survey Research Center. No identifying information was collected. This extensive survey asked participants to self-report demographic data and information regarding their work and personal factors that may contribute to stress. A portion of this instrument, with items focused on mental health diagnoses and 3 validated scales, were used in the current study. The scales were the 10-item Connor-Davidson Resilience Scale (CD-RISC) for assessing resilience [33, 34], the 12-item Linear Analog Self-Assessment (LASA) for assessing quality of life [35], and the 14-item Perceived Stress Scale (PSS) for assessing stress [36].

Each item in the CD-RISC is phrased in such a way that a higher endorsement of the statement indicates higher resilience (0 = not at all true, 1 = rarely true, 2 = sometimes true, 3 = often true, and 4 = true nearly all the time). People in the lower-resilience category tend to score individual items in the “not at all true” to “sometimes true” range; those with medium resilience tend to score more items as “often true”; and those with higher resilience tend to score items as “true nearly all the time.” Based on their CD-RISC score, participants were divided into 3 cohorts, as described in the statistical analysis section below.

### Statistical analysis

The CD-RISC score was calculated as the sum of the 10 resilience items; we included data only from participants who answered all 10 items. Possible CD-RISC scores ranged from 0 to 40. Scores were stratified into 3 groups: lower resilience (CD-RISC score <30), medium resilience (score 30–34), and higher resilience (score ≥35). These categories were based on population data [37] on CD-RISC showing a 25thpercentile score of 29, a 50th percentile score of 32, and a 75th percentile score of 36.

The overall LASA score was calculated as the average score of answered items; we included data only from those who completed at least 6 of the 12 items. Possible scores for individual items and the overall score ranged from 0 to 10. In calculating the overall LASA score, answers were reversed on the response scale as needed (eg, questions regarding frequency and severity of pain, fatigue) so that all were oriented in the direction of higher scores indicating better quality of life.

The PSS score was calculated as the sum of 14 items; we included data only from participants who answered at least 7 items. Possible PSS scores ranged from 0 to 40. In cases when not all PSS items were completed, the mean of the completed items was multiplied by 14. Answers were reversed on the response scale as needed so that higher scores indicated greater stress.

All participant characteristics are summarized with frequencies and percentages. The mean (SD) of the individual LASA items, overall LASA score, and PSS were compared among CD-RISC categories by using analysis of variance F tests. Participant characteristics, as well as the percentage of participants reporting anxiety, depression, bipolar disorder, or other mental health conditions, were each compared among CD-RISC categories with χ^2^ tests. Age was included categorically in the survey and compared using Kruskal-Wallis tests. In addition to overall comparisons, we also included pairwise comparisons using the same statistical tests noted above. The association between the noncategorized CD-RISC score with the LASA and PSS scores was quantified with Pearson correlations [38]. Adjusted associations between CD-RISC category (X) and the overall LASA score (Y) were assessed with a linear regression model, adjusting for the following covariates: age, education, gender, marital status (married vs unmarried), income, current meditator status, and race (white vs nonwhite). Adjusted associations with PSS score were assessed with the same method, whereas self-reported depression or anxiety were assessed with logistic regression models. *P* values less than .05 were considered statistically significant. All analyses were conducted with SAS (version 9.4; SAS Institute Inc).

## Results

**Fig 1:**
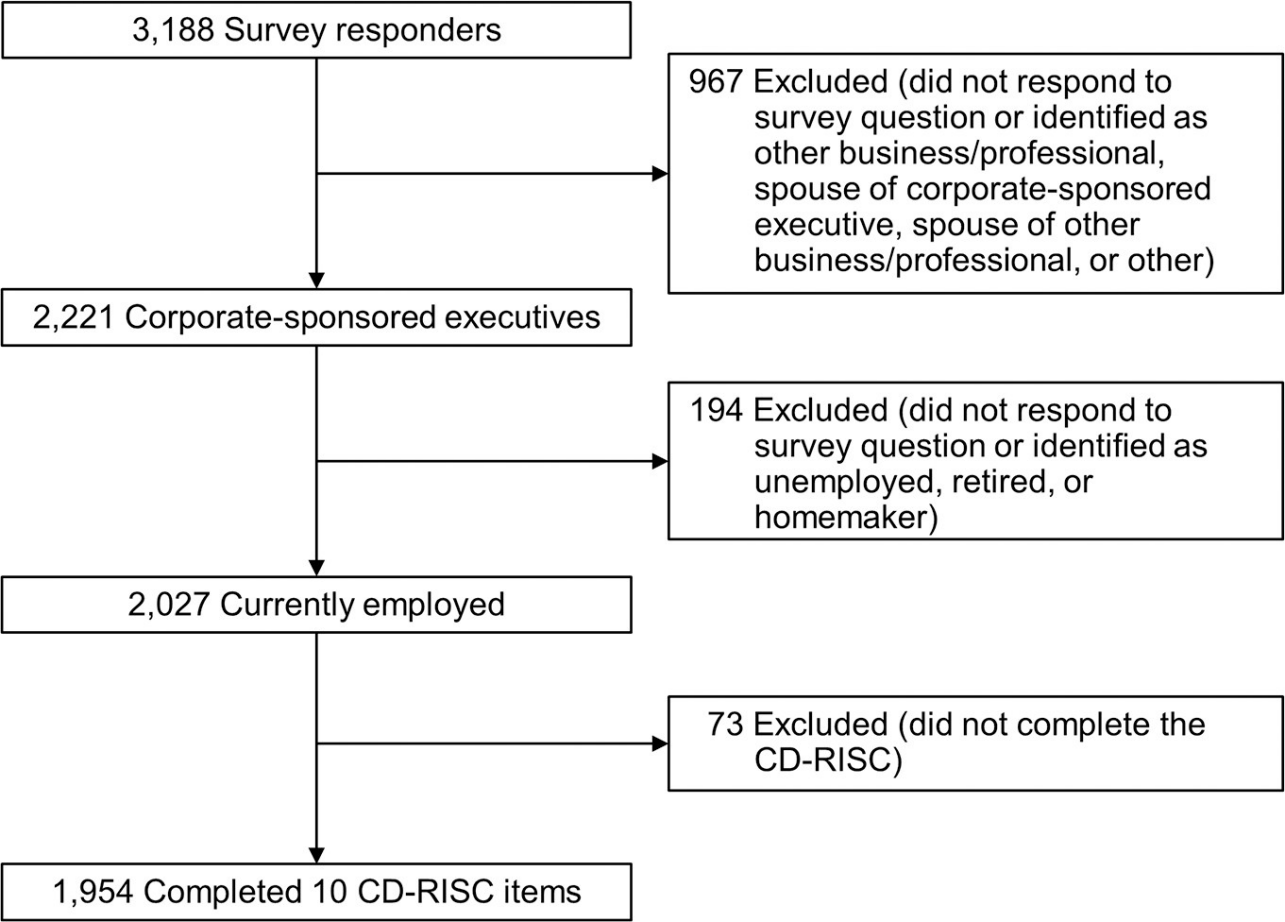
Flow diagram of patient selection. CD-RISC indicates Connor-Davidson Resilience Scale.

Of the 2,027 eligible participants in the Executive Health program, 1,954 (96.4%) completed the 10-item CD-RISC. These participants constituted the final cohort for analysis in this study (Fig 1). The majority of participants (78.3%) were male, married or in a committed relationship (89.7%), and white (95.3%). Most had either a 4-year college degree (35.6%) or a graduate or professional degree (50.6%). Most participants were 40 to 59 years old (62.5%). Household incomes of $500,000 or more were reported by 39.0%, and 58.1% had incomes ranging from $100,000 to $499,999. Fourteen percent of respondents reported that they currently practiced meditation. Table 1 summarizes the demographics of the study cohort.

**Table 1:** Participant characteristics, stratified by resilience level^a^.

		Resilience (CD-RISC), No. (%)^b^			
Characteristic	Overall, No. (%) (N = 1,954)	Lower (n = 469)	Medium (n = 671)	Higher (n = 814)	*P* Value^c^
Education					.17
High school or GED	60 (3.2)	16 (3.6)	19 (2.9)	25 (3.1)	
Some college, technical school, vocational school, or associates degree	199 (10.5)	55 (12.3)	67 (10.4)	77 (9.7)	
4-Year college degree	673 (35.6)	176 (39.4)	215 (33.3)	282 (35.4)	
Graduate or professional school	956 (50.6)	200 (44.7)	344 (53.3)	412 (51.8)	
Gender					.72
Male	1,487 (78.3)	347 (76.9)	512 (78.8)	628 (78.7)	
Female	412 (21.7)	104 (23.1)	138 (21.2)	170 (21.3)	
Age, y					.21
<40	88 (4.6)	24 (5.3)	29 (4.5)	35 (4.4)	
40–49	391 (20.6)	96 (21.2)	140 (21.6)	155 (19.4)	
50–59	796 (41.9)	198 (43.7)	267 (41.1)	331 (41.5)	
≥60	625 (32.9)	135 (29.8)	213 (32.8)	277 (34.7)	
Marital status					.17
Married or committed relationship	1,719 (89.7)	408 (88.5)	594 (90.7)	717 (89.5)	
Divorced	92 (4.8)	21 (4.6)	30 (4.6)	41 (5.1)	
Widowed	21 (1.1)	2 (0.4)	6 (0.9)	13 (1.6)	
Separated	18 (0.9)	8 (1.7)	5 (0.8)	5 (0.6)	
Never married	67 (3.5)	22 (4.8)	20 (3.1)	25 (3.1)	
Race/ethnicity					.36^d^
White	1,805 (95.3)	438 (96.5)	613 (94.7)	754 (95.0)	
Black or African American	10 (0.5)	1 (0.2)	1 (0.2)	8 (1.0)	
Asian	29 (1.5)	9 (2.0)	14 (2.2)	6 (0.8)	
Native Hawaiian or other Pacific Islander	1 (0.1)	0 (0)	1 (0.2)	0 (0)	
American Indian or Alaska Native	4 (0.2)	0 (0)	0 (0)	4 (0.5)	
Hispanic	32 (1.7)	2 (0.4)	12 (1.9)	18 (2.3)	
Other or multiple	14 (0.7)	4 (0.9)	6 (0.9)	4 (0.5)	
Household income					< .001
<$100,000	55 (2.9)	20 (4.4)	18 (2.8)	17 (2.2)	
$100,000 to $499,999	1,091 (58.1)	290 (64.4)	393 (60.9)	408 (52.0)	
≥$500,000	733 (39.0)	140 (31.1)	234 (36.3)	359 (45.8)	
Currently practicing meditation	263 (14.3)	52 (11.7)	88 (13.8)	123 (16.1)	.10

Abbreviations: CD-RISC, Connor-Davidson Resilience Scale; GED, General Education Development.

^a^ Percentages were calculated by using the total number of respondents for each question as the denominator.

^b^ Resilience groups were defined by the CD-RISC score. Lower resilience was defined as a score <30; medium resilience, 30–34; higher resilience, ≥35.

^c^ Statistically significant pairwise differences were identified only for household income (lower vs higher resilience, *P* < .001; medium vs higher resilience, *P* = .001) and current meditator status (lower vs higher resilience, *P* = .04).

^d^ Statistical test compares white vs nonwhite (all nonwhite groups combined).

Based on their scores on the 10-item CD-RISC, participants were categorized by self-reported resilience level. Participants with higher resilience (CD-RISC score ≥35) made up the largest group (n = 814 [41.7%]). Those with medium resilience (score 30–34) accounted for 34.3% of the cohort (n = 671), and those with lower resilience (score <30) were 24.0% of the cohort (n = 469). Participants with higher resilience had higher income (*P* < .001) and more commonly meditated (16.1% for higher resilience, 11.7% for lower resilience; *P* = .04). None of the remaining participant characteristics differed significantly across the CD-RISC categories.

**Fig 2:**
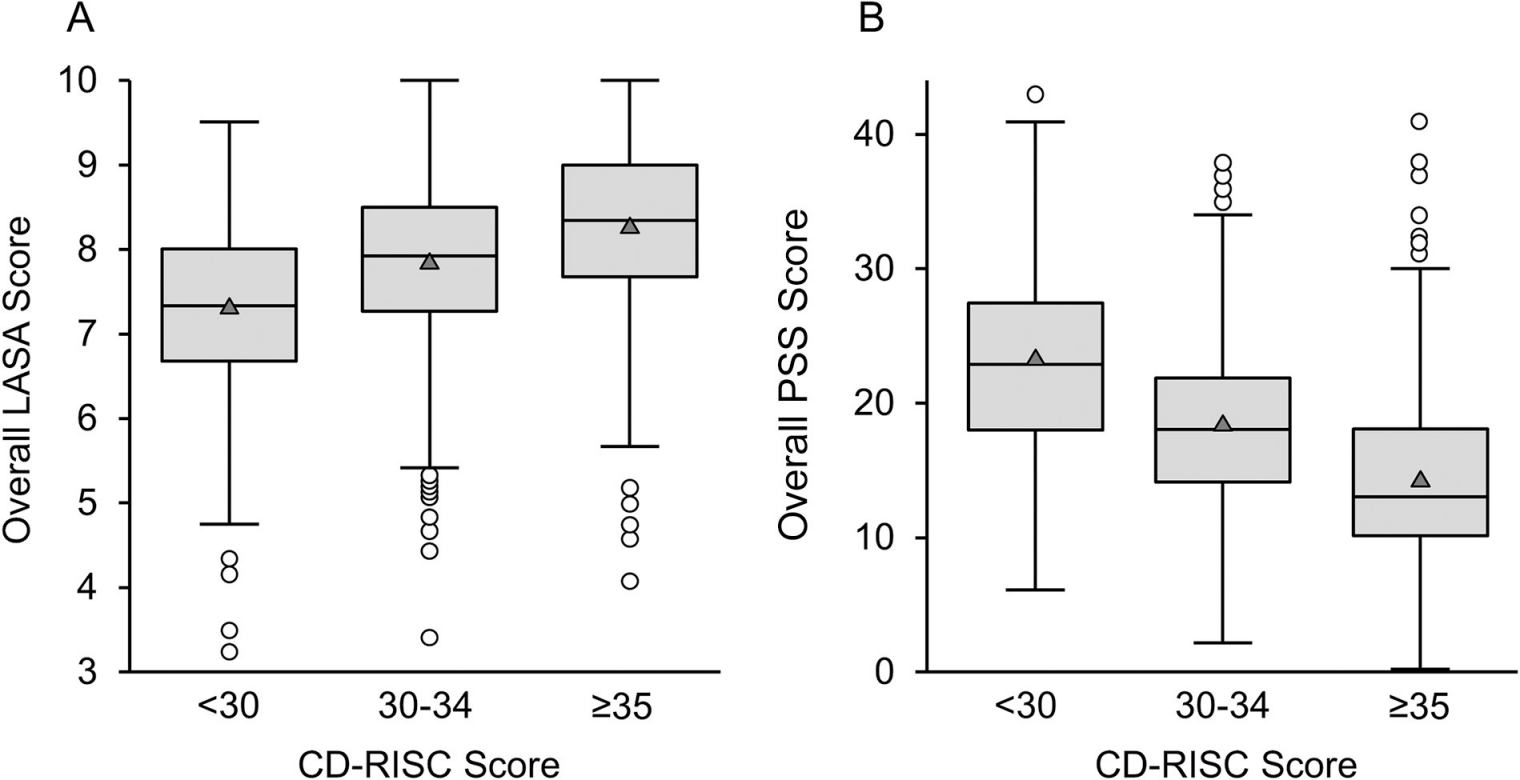
Box-and-Whisker Plots of LASA and PSS scores, Stratified by Resilience Levels. Resilience groups were defined by the CD-RISC score. Lower resilience was defined as a score <30; medium resilience, 30–34; higher resilience, ≥35. Boxes show the median (middle horizontal line), interquartile range (25^th^ percentile [Q1] and 75^th^ percentile [Q3]: lower and upper edges of box), and range; the whiskers (dashed lines) extend from the outer edges of the box to the most extreme point within a distance equal to 1.5 × (Q3-Q1); any observations extending beyond that distance are shown as individual points in the figure. A, Overall LASA score (*P* < .001 for all pairwise comparisons). B, PSS Score (*P* < .001 for all pairwise comparisons). CD-RISC indicates Connor-Davidson Resilience Scale; LASA, Linear Analogue Self-Assessment; PSS, Perceived Stress Scale.

**Fig 3:**
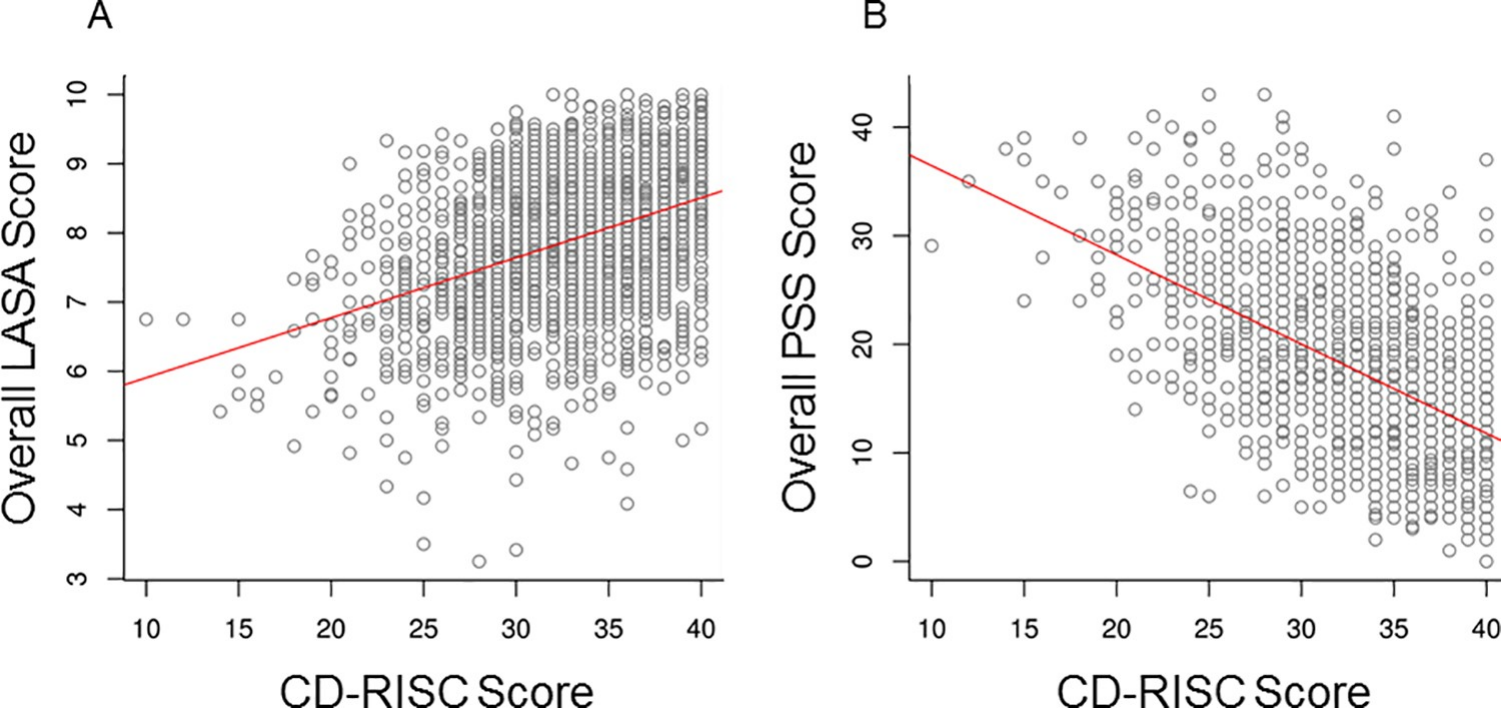
Scatterplots of association of LASA or PSS score With CD-RISC score. The estimated linear regression line is shown in red. A, Overall LASA score (correlation between LASA and CD-RISC = 0.40). B, PSS Score (correlation between PSS and CD-RISC = −0.55). CD-RISC indicates Connor-Davidson Resilience Scale; LASA, Linear Analogue Self-Assessment; PSS, Perceived Stress Scale.

For the LASA scale, the average quality-of-life scores and overall score were each positively associated with higher resilience in overall comparisons (*P* < .001) and in pairwise analyses (*P* < .05). The correlation between the overall LASA score with CD-RISC score was 0.40 (*P* < .001); medium- and high-resilience participants had average LASA scores that were 0.5 or 1.0 points higher, respectively, than scores from participants with low resilience. After adjusting for education, age, gender, white race, income, current meditator status, and marital status, the association between overall LASA and resilience was unaffected. After adjusting for these participant characteristics, average LASA scores for medium- or high-resilience participants were 0.51 and 0.92 higher, respectively, than those with low resilience (similar to the unadjusted differences reported above; *P* < .001). Conversely, the PSS score was negatively associated with resilience in overall comparisons (*P* < .001) and in pairwise analyses (*P* < .05), with average PSS scores being 23.2, 18.3, and 14.1 in the lower-, medium-, and higher-resilience categories, respectively (correlation, −0.55; *P* < .001). The adjusted association between PSS and resilience was unaffected. After adjusting for the participant characteristics, average PSS scores were 5.0 and 9.2 lower for those with medium or high resilience, respectively, compared with participants with low resilience (similar to the unadjusted differences of 4.9 and 9.1; *P* < .001). Table 2 summarizes the stress and well-being measures, Fig 2A and 2B illustrate the distribution of the overall LASA and PSS by resilience level categories, and Fig 3A and 3B illustrate the correlations of LASA and PSS with resilience on a noncategorized scale.

**Table 2:** LASA and PSS scores, stratified by resilience levels.

			Resilience (CD-RISC)^a^				
Survey	Definition	Overall (N = 1,954)	Lower (n = 469)	Medium (n = 671)	Higher (n = 814)	*P* Value	Pairwise *P* Value^b^
LASA score, mean (SD)							
Quality of life	10 = As good as it can be	8.4 (1.2)	7.9 (1.3)	8.3 (1.1)	8.7 (1.2)	< .001	A, B, C
Mental well-being	10 = As good as it can be	8.5 (1.2)	7.8 (1.2)	8.4 (1.1)	8.9 (1.0)	< .001	A, B, C
Physical well-being	10 = As good as it can be	7.6 (1.5)	7.0 (1.5)	7.6 (1.3)	7.9 (1.4)	< .001	A, B, C
Emotional well-being	10 = As good as it can be	8.2 (1.4)	7.3 (1.5)	8.1 (1.2)	8.6 (1.2)	< .001	A, B, C
Spiritual well-being	10 = As good as it can be	7.9 (1.6)	7.2 (1.6)	7.8 (1.6)	8.4 (1.4)	< .001	A, B, C
Level of social activity	10 = As good as it can be	7.9 (1.6)	7.2 (1.8)	7.9 (1.5)	8.2 (1.6)	< .001	A, B, C
Frequency of pain	10 = Constant pain	2.5 (2.5)	2.7 (2.5)	2.6 (2.6)	2.3 (2.5)	.03	B, C
Severity of pain	10 = Pain as bad as you can imagine	1.9 (1.9)	2.1 (1.9)	2.0 (2.0)	1.7 (1.8)	< .001	B, C
Fatigue	10 = Constant tiredness	3.2 (2.3)	4.1 (2.2)	3.2 (2.1)	2.7 (2.3)	< .001	A, B, C
Support from friends and family	10 = Highest level of support	8.1 (1.9)	7.5 (1.8)	8.0 (2.0)	8.6 (1.7)	< .001	A, B, C
Financial concerns	10 = No concerns	7.7 (2.4)	7.1 (2.5)	7.8 (2.3)	8.1 (2.4)	< .001	A, B, C
Legal concerns	10 = No concerns	8.0 (2.7)	7.4 (2.7)	7.9 (2.7)	8.4 (2.6)	< .001	A, B, C
Overall LASA score^c^		7.9 (1.0)	7.3 (1.0)	7.8 (1.0)	8.3 (1.0)	< .001	A, B, C
PSS, mean (SD), score^d^		17.7 (7.2)	23.2 (6.9)	18.3 (6.1)	14.1 (6.1)	< .001	A, B, C

Abbreviations: CD-RISC, Connor-Davidson Resilience Scale; LASA, Linear Analogue Self-Assessment; PSS, Perceived Stress Scale.

^a^ Resilience groups were defined by the CD-RISC score. Lower resilience was defined as a score <30; medium resilience, 30–34; higher resilience, ≥35.

^b^ Referring to significant (*P* < .05) pairwise comparisons. A: Groups 1 vs 2, B: Groups 1 vs 3, C: Groups 2 vs 3.

^c^ The average score was calculated after orienting each LASA item so that a higher score indicated a better quality of life. Of the 1,954 survey respondents, 1,769 completed all 12 LASA items and 184 completed 6–11 items.

^d^ A higher score indicated more stress. Of the 1,954 survey respondents, 1,810 completed all 14 PSS items and 91 completed 7–13 items.

The percentage of participants indicating a history of depression, anxiety, or bipolar disorder was significantly higher among those reporting lower resilience in overall comparisons (*P* < .001) and in pairwise analyses (*P* < .05). As compared with those with low resilience, the odds ratios (ORs) for depression were 0.45 and 0.21 for those with medium and high resilience, respectively. Adjusting for education, gender, age, marital status, income, current meditator status, and white race had little effect on these results for depression (adjusted ORs, 0.47 and 0.20; *P* < .001 for both). Results were similar for anxiety; as compared with those with low resilience, the ORs for anxiety were 0.53 and 0.30 for those with medium and high resilience, respectively. The adjusted ORs for anxiety were 0.53 and 0.30 (*P* < .01 for both). The number of individuals reporting bipolar disorder was too low for adjusted analyses. Table 3 summarizes the mental health diagnoses self-reported by the study cohort.

**Table 3:** Self-reported mental health diagnoses, stratified by resilience levels.

		Resilience (CD-RISC), No. (%)^a^^,^^b^				
Diagnosis	Overall, No. (%)^a^	Lower (n = 469)	Medium (n = 671)	Higher (n = 814)	*P* Value	Pairwise *P* Value^c^
Anxiety	282/1,900 (14.8)	113/457 (24.7)	97/654 (14.8)	72/789 (9.1)	< .001	A, B, C
Depression	190/1,901 (10.0)	89/456 (19.5)	64/654 (9.8)	37/791 (4.7)	< .001	A, B, C
Bipolar disorder	10/1,883 (0.5)	4/446 (0.9)	5/649 (0.8)	1/788 (0.1)	< .001	B, C
Other mental health disorders	23/994 (2.3)	8/243 (3.3)	8/324 (2.5)	7/427 (1.6)	.47	…

Abbreviation: CD-RISC, Connor-Davidson Resilience Scale.

^a^ The N varies for each question because respondents skipped some survey items.

^b^ Resilience groups were defined by the CD-RISC score. Lower resilience was defined as a score <30; medium resilience, 30–34; higher resilience, ≥35.

^c^ Referring to significant (*P* < .05) pairwise comparisons. A: Groups 1 vs 2, B: Groups 1 vs 3, C: Groups 2 vs 3.

## Discussion

This large cross-sectional survey of executives showed that participants with higher resilience reported a higher quality of life and perceived less stress than those with medium or lower resilience. The association was unaffected by adjustments for education, age, gender, race, marital status, income, and current meditator status. Further, the self-reported history of depression, anxiety, and bipolar disorder was significantly different across levels of resilience, with the lower-resilience cohort reporting a 4-fold higher prevalence of depression compared with the higher-resilience cohort. A moderately positive correlation was observed between resilience and quality of life, and a moderately negative correlation was observed between resilience and perceived stress.

Our results are consistent with previous studies that assessed the association of resilience with stress and well-being measures, psychological distress, and mental health diagnoses. Our findings are supported by the prior studies that have been conducted in various patient groups, including renal transplant recipients [39], patients undergoing hematopoietic stem cell transplant and their relatives [40], patients with cancer [41, 42], patients with head and neck cancer [43], patients with digestive system cancer [44–46], trauma patients [47], patients with rare health conditions [48], and patients with spinal cord injury [49].

Two groups of studies have evaluated the positive association between resilience and better mental health among healthy adults. The first set of studies, predominantly of university students, showed that higher resilience was consistently associated with lower psychological distress and better mental health [50–57]. The second group of studies evaluated the effect of resilience in specific demographic groups of adults. Among the professional groups, the most commonly studied workers were in health care and included nurses, physicians and midlevel practitioners, and health professionals in a critical care setting [24–28, 58, 59]. Others have studied refugees [60], veterans [61], tennis players [62], Spanish athletes [63], couples with infertility undergoing in vitro fertilization [64, 65], and healthy adults [66]. Although most of these studies were small and had narrow demographic groups, the association between resilience and better mental health was consistent.

Our study is novel in that it explores the role of resilience as a protective factor in the corporate setting. The large inverse association of resilience with anxiety and depression was noteworthy, with an almost 3-fold higher prevalence of anxiety and a 4-fold higher prevalence of depression in the lower-resilience group compared with the higher-resilience group. In a previous study, designed as an online survey of workers, lower resilience similarly had a strong association with a higher prevalence of depression for environments with low and high work strain [30]. Given the high prevalence of stress in the corporate environment and mental health diagnoses in this executive population, promoting resilience at workplaces through organizational and individual interventions may be a strategy that helps buffer the negative consequences of workplace stress [67–72].

Our study has several limitations, including the cross-sectional design, self-reported outcomes, and predominantly male cohort. The cross-sectional design shows only associations but not causative relationships, and our ability to discern the direction of association is limited. Self-reported outcomes affect the validity of the results. The lack of demographic diversity limits the generalizability of our study findings.

In summary, we report that high resilience was associated with significant and meaningful differences in stress and well-being measures and mental health diagnoses among corporate executives. The large differences noted in our study suggest that interventions to enhance resilience, at the individual and organizational level, may help mitigate negative consequences of work-related stress.

## Supporting information

S1 Data Raw survey data. (CSV)
